# UST2/475: Are Parents Appreciating Medical Web Sites? Results from an Italian survey

**DOI:** 10.2196/jmir.1.suppl1.e128

**Published:** 1999-09-19

**Authors:** V Currò, A D'Atri, V Mauro, A Bernabei, S Buonuomo

**Affiliations:** ^1^Luiss Guido Carli University, CeRSILRomaItaly

**Keywords:** Pediatrics, Parents, Internet, Medical Information

## Abstract

**Introduction:**

The growth of the number of Internet users leads to an increasing demand for information quality control, especially when information is not oriented to healthcare professionals but to patients and/or general citizens. Even if several surveys have been done to evaluate the kind of access to medical information the relevance of this phenomenon in the families does not seems adequately investigated. In fact navigators (see Oct.98 GVU survey) are mainly (70%) without sons and access to medical information over fifty. The goal of this research is to evaluate the number and characteristics of Italian parents that could be interested to medical webs. This study is the first step to the main goal of evaluating paediatrics information quality provided by Web sites aimed to educate parents.

**Methods:**

The survey, done in two Italian cities, involved 284 families having access to a Internet, and responding to questionnaires distributed and collected (May 24th to June 10th) mainly in primary schools. Parents were asked to answer about the frequency and location of access, whether they visited medical sites and the usefulness of them. To evaluate its readability and to select the size of the sample, this questionnaire was tested with 79 parents, during a one week pilot phase carried out in the outpatient paediatrics department of Policlinico A. Gemelli, Rome. To respect data privacy, questions about sensible personal data were not included and it was filled in an anonymous way. RESULTS: The characteristics of the sample are the following ones.-41% have higher school education and 56% are graduated. -65% are employees and 28% are free-lance. -the average age for the fathers is 42.8 years (29 to 58) and for the mothers is 39.7 years (28 to 53); -48% are mothers and 52% are fathers;-the majority of families (53%) has 2 sons and 31% of them has 1 son. The usage of internet shows the following Results:

- 10% connect mainly from both office and home, 56% connect mainly from the office, and 29% mainly from home
- 25% has a daily connection, 34% more than once a week, 17% less than once a week and 16% once a month
- 35% has visited medical sites (40% of the fathers and 31% of the mothers) and, among them, 56% found medical sites useful and 26% very useful

**Discussion:**

The usage of Internet at home is less frequent than from the office: this shows the minor diffusion of Internet in the Italian homes, probably because of the high communication costs. A very interesting result is the high number of internet users accessing medical sites (about one third); furthermore the great majority of the parents navigating through medical sites find this information useful or very useful. Another interesting results is the fact that our survey shows a greater interest in medical sites from fathers with respect to mothers (GVU survey stated a greater and more frequent access of women to medical information).

